# Prediction of the Effects of Nonsynonymous Variants on SARS-CoV-2 Proteins

**DOI:** 10.12688/f1000research.72904.1

**Published:** 2022-01-06

**Authors:** Boon Zhan Sia, Wan Xin Boon, Yoke Yee Yap, Shalini Kumar, Chong Han Ng

**Affiliations:** 1 Faculty of Information Science and Technology, Multimedia University, Bukit Beruang, Melaka, 75450, Malaysia

**Keywords:** SARS-CoV-2, nonsynonymous mutation, co-mutation, COVID-19

## Abstract

**Background:** SARS-CoV-2 virus is a highly transmissible pathogen that causes COVID-19. The outbreak originated in Wuhan, China in December 2019. A number of nonsynonymous mutations located at different SARS-CoV-2 proteins have been reported by multiple studies. However, there are limited computational studies on the biological impacts of these mutations on the structure and function of the proteins.

**Methods**: In our study nonsynonymous mutations of the SARS-CoV-2 genome and their frequencies were identified from 30,229 sequences. Subsequently, the effects of the top 10 nonsynonymous mutations of different SARS-CoV-2 proteins were analyzed using bioinformatics tools including co-mutation analysis, prediction of the protein structure stability and flexibility analysis, and prediction of the protein functions.

Results: A total of 231 nonsynonymous mutations were identified from 30,229 SARS-CoV-2 genome sequences. The top 10 nonsynonymous mutations affecting nine amino acid residues were ORF1a nsp5 P108S, ORF1b nsp12 P323L and A423V, S protein N501Y and D614G, ORF3a Q57H, N protein P151L, R203K and G204R. Many nonsynonymous mutations showed a high concurrence ratio, suggesting these mutations may evolve together and interact functionally. Our result showed that ORF1a nsp5 P108S, ORF3a Q57H and N protein P151L mutations may be deleterious to the function of SARS-CoV-2 proteins. In addition, ORF1a nsp5 P108S and S protein D614G may destabilize the protein structures while S protein D614G may have a more open conformation compared to the wild type.

Conclusion: The biological consequences of these nonsynonymous mutations of SARS-CoV-2 proteins should be further validated by in vivo and in vitro experimental studies in the future.

## Introduction

A new coronavirus disease known as COVID-19, caused by severe acute respiratory syndrome coronavirus 2 (SARS-CoV-2), was first reported in Wuhan, China in December 2019. ^1^ SARS-CoV-2 is a positive-sense single stranded RNA virus with a helical nucleocapsid. The genome size of SARS-CoV-2 is about 30 kilobases. There are 11 protein-coding genes from the SARS-CoV-2 genome including four structural genes (spike (S), envelope (E), membrane (M), and nucleocapsid (N) genes) and seven nonstructural genes (ORF1ab, ORF3a, ORF6, ORF7a, ORF7b, ORF8 and ORF10). ^2^

The SARS-CoV-2 virus can rapidly mutate to bypass the immune response of the host. ^3^ These mutations can be synonymous, nonsynonymous, deletions, insertions, or others. Nonsynonymous mutations are expected to have a greater impact than synonymous mutations since nonsynonymous mutations affect the amino acid sequences of a protein, subsequently they may change their structures and functions. According to Kim et al. (2020), a total of 767 synonymous and 1352 nonsynonymous mutations have been identified from SARS-CoV-2 genomes. ^4^ In another study, a total of 119 SNPs were identified using 11,183 SARS-CoV-2 genomes, in which there were 74 nonsynonymous mutations and 43 synonymous mutations. ^5^ From a study on the analysis of nonsynonymous mutations in structural proteins of SARS-CoV-2, it has been shown that S and N proteins have higher mutation rate per gene compared to that of E and M proteins. ^6^ However, the biological consequences of these mutations on the functions and structures of SARS-CoV-2 proteins remain unclear. In our study, computational analysis of the nonsynonymous mutations of SARS-CoV-2 proteins were performed using different bioinformatics tools including co-mutation analysis, protein structure stability and flexibility analysis, and protein function analysis to predict the effects of the mutations on the structures and functions of proteins.

## Methods

### Sequences and structures retrieval

The SARS-CoV-2 genomes data were downloaded from GISAID database (Global Initiative on Sharing All Influenza Data, RRID:SCR_018251). ^7^ In this study, a total number of 30,229 SARS-CoV-2 virus genomes data with collection dates ranging from 2020-01-01 to 2021-03-21 were retrieved. To make sure that only high-quality sequences were used, the filters including complete genome, high coverage and patient status, excluding low coverage were applied. The reference strain NC_045512.2 with a total number of 29903 bases was retrieved from NCBI database (NCBI, RRID:SCR_006472). The wild type protein structures obtained from RCSB PDB (Research Collaboratory for Structural Bioinformatics Protein Data Bank, RRID:SCR_012820) are listed in Table 1. ^8^ Since N protein R203 and G204 are located at a disordered region which does not have a well-defined three-dimensional structure, no experimental structural data was available for the prediction analysis. A predicted model of N protein model ( QHD43423, estimate TM-score = 0.97) generated with D-I-TASSER/C-I-TASSER pipeline was used. ^9^

**Table 1.** SARS-CoV-2 protein structures used in this study.

Protein	Nucleotide changes	Amino acid changes	Template structure (PDB ID)
ORF1a nsp5	C10376T	P108S	7KPH
ORF1b nsp12	C14408T	P323L	6YYT
	C14708T	A423V	6YYT
S	A23063T	N501Y	7A92
	A23403G	D614G	7A92
ORF3a	G25563T	Q57H	6XDC
N	C28725T	P151L	6VYO
	G28881A	R203K	QHD43423
	G28882A	R203K	QHD43423
	G28883C	G204R	QHD43423

### Multiple sequence alignment of SARS-CoV-2 genomes

Multiple sequence alignment (MSA) was performed using rapid calculation in MAFFT (MAFFT, version 7.467, RRID:SCR_011811) which supports alignment for more than 20,000 sequences. ^10^ After all SARS-CoV-2 sequences were aligned to the reference genome, the multiple sequence alignment file was visualized under MEGA X software, version 10.2.5 build 10210330 ( MEGA Software, RRID:SCR_000667).

### Identification of nonsynonymous mutations and the statistics of the mutation in the SARS-CoV-2 proteins

The 11 different coding sequences were extracted from these 30,229 strains according to their genomic positions in the reference strain (fasta file format) in NCBI, which is NC_045512.2. Inappropriate sequences of base calling errors, “N” unresolved nucleotides, and undefinable gaps were omitted. Then, the frequency and number of nonsynonymous mutations in these 30,229 strains were identified using a Python script.

### Co-mutation analysis of SARS-CoV-2 proteins

The concurrence ratio of each nonsynonymous mutation in the SARS-CoV-2 genome was determined using GESS database (The Global Evaluation of SARS-CoV-2/hCoV-19 Sequences, RRID:SCR_021847) ^11^ derived from GISAID web server. The concurrence search used for the analysis of the concurrence ratio in the top 10 nonsynonymous mutations is listed in Table 2. The frequency for each SNV in the concurrence search is greater than 0.1%. The chord diagram for co-mutations of nonsynonymous mutations in the SARS-CoV-2 genome was generated using Circos table viewer (Circos, RRID:SCR_011798). ^12^

**Table 2.** Concurrence ratio of top 10 nonsynonymous mutations in SARS-CoV-2 proteins.

Coding region and amino acid change		ORF1a nsp5 P108S	ORF1b nsp12 P323L	ORF1b nsp12 A423V	S protein N501Y	S protein D614G	ORF3a Q57H	N protein P151L	N protein R203K	N protein R203K	N protein G204R
Coding region and amino acid change	Nucleotide change	C10376T	C14408T	C14708T	A23063T	A23403G	G25563T	C28725T	G28881A	G28882A	G28883C
**ORF1a nsp5 P108S**	**C10376T**	0	99.9	98.3	0.2	99.9	4.6	97.2	91.6	91.6	91.7
**ORF1b nsp12 P323L**	**C14408T**	99.9	0	99.9	98.8	99.8	99.7	99.9	99.7	99.8	99.8
**ORF1b nsp12 A423V**	**C14708T**	98.3	99.9	0	0.1	99.9	0.8	98.3	98.6	98.6	98.6
**S protein N501Y**	**A23063T**	0.2	98.8	0.1	0	99.2	25.7	0.2	67.3	65.6	66.7
**S protein D614G**	**A23403G**	99.9	99.8	99.9	99.2	0	99.9	100	99.6	99.7	99.7
**ORF3a Q57H**	**G25563T**	4.6	99.7	0.8	25.7	99.9	0	1.1	0.4	0.3	0.2
**N protein P151L**	**C28725T**	97.2	99.9	98.3	0.2	100	1.1	0	98.1	98.1	98.1
**N protein R203K**	**G28881A**	91.6	99.7	98.6	67.3	99.6	0.4	98.1	0	99.9	99.9
**N protein R203K**	**G28882A**	91.6	99.8	98.6	65.6	99.7	0.3	98.1	99.9	0	99.9
**N protein G204R**	**G28883C**	91.7	99.8	98.6	66.7	99.7	0.2	98.1	99.9	99.9	0

### Prediction of mutation effect on protein stability and flexibility

To predict the effects of the mutations on the stability and flexibility of the protein structure, the protein structures were analyzed with DynaMut server (DynaMut, RRID:SCR_021849). ^13^ The free energy change between the wild type and mutant protein structure (ΔΔG) predicts the status of protein stability, in which the values of ΔΔG above zero indicate a good stabilization while any values below zero or negative indicate a destabilizing outcome. The difference in entropic energy between the wild type and mutant structures (ΔΔS _Vib_ ENCoM) predicts the status of protein flexibility, in which the values of ΔΔS _Vib_ ENCoM above zero indicate an increase in flexibility while any values below zero or negative indicate a decrease in flexibility.

### Prediction of mutation effect on protein function

SIFT 4G (Sorting Tolerant From Intolerant For Genomes, RRID:SCR_021850) ^14^ and PROVEAN (Protein Variation Effect Analyzer, RRID:SCR_002182) ^15^ were used to predict the deleteriousness of the nonsynonymous single nucleotide polymorphisms (nsSNPs) on SARS-CoV-2 protein structure. SIFT 4G predicts the effects of the mutations based on the sequence conservation and amino acid properties. For SIFT 4G analysis, gene annotation files (GTF), fasta files containing the SARS-CoV-2 genome sequences, and a variant call format file (VCF) comprising all the SNP of SARS-CoV-2 were obtained. After that, the SARS-CoV-2 genome database, built with the SIFT 4G algorithm, was created. Lastly, SIFT 4G annotator was applied to annotate the VCF file with SARS-CoV-2 genome database. Mutations with a SIFT 4G score of less than 0.05 were considered deleterious. PROVEAN predicts the effects of the mutations based on the principle of alignment-based score. For PROVEAN analysis, the amino acid sequence along with the amino acid variation were processed in the PROVEAN server to get the prediction result. Mutations with a value less than −2.5 were considered as deleterious.

## Results

### The statistics of nonsynonymous mutations in SARS-CoV-2 proteins

**Figure 1.**
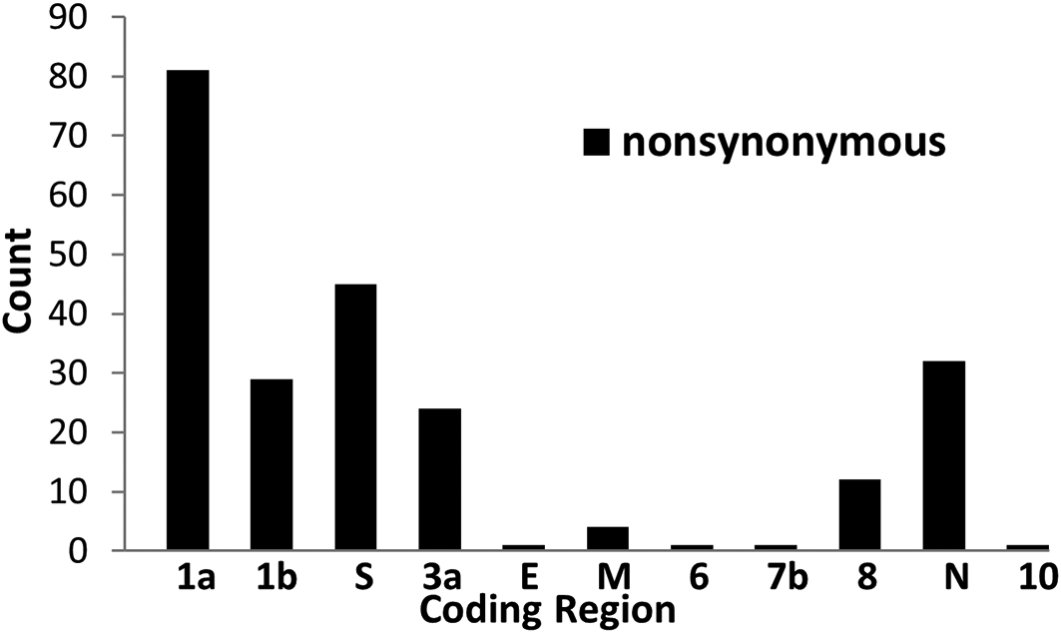
The numbers of nonsynonymous mutations in 11 coding sequences of SARS-CoV-2 proteins.

From the multiple alignment analysis, we identified 231 nonsynonymous mutations from 30,229 SARS-CoV-2 genome sequences. Figure 1 shows the numbers of the nonsynonymous mutations found in 11 coding sequences of SARS-CoV-2 proteins. ORF1a has the highest numbers of nonsynonymous mutations, followed by S protein and N protein. The top 10 nonsynonymous mutations affecting 9 amino acids residues including ORF1a nsp5 P108S, ORF1b nsp12 P323L and A423V, S protein N501Y and D614G, ORF3a Q57H, N protein P151L, R203K and G204R are shown in Table 3.

**Table 3.** Top 10 nonsynonymous mutations of SARS-CoV-2 proteins.

Protein	Nucleotide changes	Amino acid changes	Frequency
ORF1a nsp5	C10376T	P108S	4024
ORF1b nsp12	C14408T	P323L	27953
	C14708T	A423V	3988
S	A23063T	N501Y	4218
	A23403G	D614G	28022
ORF3a	G25563T	Q57H	5274
N	C28725T	P151L	4007
	G28881A	R203K	18116
	G28882A	R203K	18092
	G28883C	G204R	18090

### Co-mutation analysis of SARS-CoV-2 proteins

**Figure 2.**
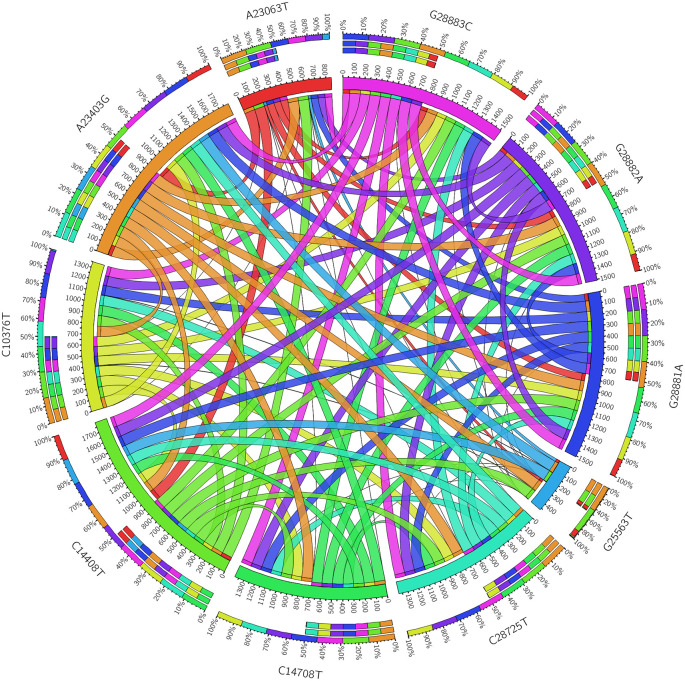
Visualization of co-mutation in top 10 nonsynonymous mutations in SARS-CoV-2 proteins.

Some nonsynonymous mutations may be random and have no or little biological impact on viral transmission and pathogenesis. If a single nonsynonymous mutation co-mutates with other mutations, they may evolve together and interact functionally. To study co-mutation between different nonsynonymous mutations, the concurrence ratio of co-mutations in the top 10 nonsynonymous mutations was retrieved from GESS database website as shown in Table 2. The visualization of co-mutations in the top 10 nonsynonymous mutations generated with Circos table view is shown in Figure 2. In this chord diagram, connection ribbons represent co-mutations and each ribbon between row and column segments represents the value of concurrence ratio in each top 10 nonsynonymous mutations. Single colours encoded in circular arranged segments represent its own specific mutation whereas rainbow colours represent co-mutation in each mutation. The size of circular arrangement segments is proportional to the total value of concurrence ratio in a row or column. The circular size segment of ORF3a Q57H (G25563T) with the smallest segment size means the total value of concurrence ratio in row or column of ORF3a Q57H (G25563T) having the lowest concurrence ratio. A high concurrence ratio shows high co-mutation between each mutation with thicker ribbon size. S protein D614G (A23403G) with all other nine nonsynonymous mutations had concurrence ratios greater than 99%. On the other hand, low concurrence ratio shows low co-mutation with thinner ribbon size, for example, mutation ORF3a Q57H (G25563T) had the lowest concurrence ratio, only having a high concurrence ratio with S protein D614G (A23403G) and ORF1b nsp12 (P323L) C14408T, the top 2 nonsynonymous mutations which were present in more than 90% of the reported sequences.

### Prediction of mutation effect on protein stability and flexibility

Table 4 summarizes the results of predicted effects of mutations on protein stability and flexibility obtained from DynaMut. Only two mutations, namely ORF1a nsp5 P108S and S protein D614G were predicted to be destabilizing with ΔΔG values of −0.288 and −0.072, respectively. For the prediction of protein flexibility, only S protein D614G was predicted to have an increase in flexibility with an ΔΔS _Vib_ ENCoM value of 0.523.

**Table 4.** Prediction of nonsynonymous mutation effect on SARS-CoV-2 proteins stability.

Protein	Mutation	ΔΔG (kcal/mol)	Prediction outcome	ΔΔS _Vib_ ENCoM (kcal.mol ^−1^.K ^−1^)	Molecule flexibility
ORF1a nsp5	P108S	−0.288	Destabilizing	−0.208	Decrease
ORF1b nsp12	P323L	1.784	Stabilizing	−0.432	Decrease
	A423V	0.776	Stabilizing	−0.348	Decrease
S	N501Y	0.013	Stabilizing	−0.088	Decrease
	D614G	−0.072	Destabilizing	0.523	Increase
ORF3a	Q57H	0.275	Stabilizing	−0.160	Decrease
N	P151L	1.111	Stabilizing	−0.325	Decrease
	R203K	0.749	Stabilizing	−0.107	Decrease
	G204R	1.064	Stabilizing	−2.522	Decrease

### Prediction of mutation effect on protein function

The prediction results of nonsynonymous mutations in the SARS-CoV-2 proteins using SIFT 4G and PROVEAN are shown in Table 5. SIFT 4G functional missense mutation score predicted that the P108S mutation in ORF1a nsp5 was deleterious (score 0.00) while four mutations S protein D614G, ORF3a Q57H, N protein R203K and G204R were tolerated (>0.05). However, the SIFT 4G results of ORF1b nsp12 P323L and A423V, S protein N501Y and N protein P151L mutations cannot be obtained due to missing data in the Ensembl database. For the PROVEAN score, three nonsynonymous mutations, namely ORF1a nsp5 P108S, ORF3a Q57H and N protein P151L were predicted to be deleterious (score < −2.5). However, six nonsynonymous mutations, namely ORF1b nsp12 P323L and A423V, S protein N501Y and D614G, N protein R203K and G204R were predicted to be neutral (score > −2.5).

**Table 5.** Prediction of nonsynonymous mutation effect on SARS-CoV-2 proteins function.

Protein	Mutation	SIFT 4G	Provean
ORF1a nsp5	P108S	0.00 (deleterious)	−3.71 (deleterious)
ORF1b nsp12	P323L	-	−0.91 (neutral)
	A423V	-	1.21 (neutral)
S	N501Y	-	−0.09 (neutral)
	D614G	1.00 (tolerated)	0.60 (neutral)
ORF3a	Q57H	0.61 (tolerated)	−3.29 (deleterious)
N	P151L	-	−4.93 (deleterious)
	R203K	0.11 (tolerated)	−1.60 (neutral)
	G204R	0.08 (tolerated)	−1.66 (neutral)

## Discussion

The top 10 nonsynonymous mutations of SARS-CoV-2 identified from 30,229 SARS-CoV-2 genome sequences were further analyzed with co-mutation analysis, prediction of the protein structure stability and flexibility analysis, and prediction of the protein function analysis. To determine if two nonsynonymous mutations of SARS-CoV-2 proteins co-mutate, concurrence ratio was calculated. Many nonsynonymous mutations showed a high concurrence ratio, suggesting these mutations may evolve together and interact functionally. The top 2 nonsynonymous mutations, S protein D614G and ORF1b nsp12 P323L (as known as RNA-dependent RNA polymerase) showed very high concurrence ratio with other mutations since they emerged in the early phase of the pandemic. Previously it has been shown that S protein D614G co-evolved with ORF1b nsp12 P323L. ^16^ The combination of both mutations may enhance viral fitness based on epidemiological data, although the molecular mechanisms of this evolutionary advantage remain elusive. ^16^ In another study, it has been predicted that multiple SARS-CoV-2 genes may have epistatic interactions linked to viral fitness. ^17^ The effects of a mutation can be neutral, harmful, or beneficial to the virus. It is expected that most single mutations have a small effect on viral fitness. It remains an arduous task to associate a specific phenotype with a single viral mutation since it is possible that a specific phenotype is contributed to by the effects of multiple mutations.

There are huge numbers of single nucleotide polymorphisms (SNPs) present in the SARS-CoV-2 genome, hence evaluating the biological functions of all SNPs using experimental approaches is not feasible. Therefore, prediction of the effects of SNPs allows us to prioritize variants which may have some significant biological functions. Our study used the meta-prediction approach to perform functional predictions of nonsynonymous mutations to minimize the false positive rate. When two or three tools are combined, the prediction accuracy increases and reaches greater performance, however, the sensitivity is subsequently decreased as more tools are combined. ^18^

Of all these nine protein mutations, only two mutations namely ORF1a nsp5 P108S and S protein D614G were predicted to reduce their stability whereas only S protein D614G may have more a flexible conformation compared to the wild type. S protein binds to human ACE2 receptors to gain access to the host cell. ^19^ D614G mutation is found at S1 domain which is involved in receptor binding. ^20^ Two independent studies of S protein D614G mutant structures derived from cryo-electron microscopy analysis has demonstrated that the G614 mutant adopts a more open conformation compared to D614 wild type. ^21^^,^^22^ Interestingly, an *in vitro* study has shown that S protein D614G mutation may enhance virus infectivity by promoting the packing of S protein into the virion, not by enhancing the binding of S protein to the ACE2 receptor. ^23^ On the other hand, ORF1a nsp5, also known as 3C-like protease is responsible for cleaving viral polypeptides during replication. ^2^ A study by Abe et al., (2021) has showed that ORF1a nsp5 protein P108S mutation diminished its activity, possibly leading to a reduction in disease severity. ^24^

Since the protein function depends directly on the three-dimensional structure of the protein, we wanted to see if these mutations may affect the function of the protein using SIFT 4G and PROVEAN prediction tools. The PROVEAN tool is applicable for all organisms. SIFT4G, instead of SIFT was used since it allows us to build a SARS-CoV-2 genome database with variant annotation. Interestingly ORF1a nsp5 protein P108S mutation was the only mutation found to be deleterious from both SIFT4G and PROVEAN functional analysis. Together with the DynaMut stability result, it has been demonstrated that this mutation may be harmful to the virus itself, and can be less damaging to the human host as reported by Abe et al., (2021). ^24^ On the other hand, ORF3a Q57H and N protein P151L mutations are predicted to be deleterious by the PROVEAN tool only. ORF 3a is an ion channel (viroporin) which is involved in viral egress steps through lysosomal trafficking. ^25^^,^^26^ ORF3a Q57H mutation not only causes a change in amino acid in ORF3a, but also produces a truncated ORF3b due to the overlapping protein-coding sequences shared by ORF3a and ORF3b. ^27^ However, there are conflicting results about the effect of the ORF3a Q57H mutation on the human host immune response. ^27^^,^^28^ N protein is involved in the liquid-liquid phase separation for the viral genome packaging. ^29^ N protein P151L mutation is located at the RNA binding domain. It has been proposed that this mutation may disrupt the protein-drug interaction. ^30^ Although another two N protein mutations, R203K and G204R were not predicted to be deleterious in our study, they have been identified in the alpha variant, B.1.1.7, gamma variant, P.1, lambda variant, C.37 and omicron variant, BA.1/B.1.1.529. ^31^ While N protein, T205I mutation has been reported in the beta variant, B.1.351 and Mu variant, B.1.621. ^31^ More recently, another N protein mutation, R203M has been reported in the delta variant, B.1.617.2. ^31^ Interestingly mutants with N protein S202R or R203M mutations can pack more RNA material compared to the wild type based on *in vitro* studies. ^32^ These observations and experimental results suggest that N protein residues, S202, R203, G204 and T205 may play some role on viral RNA replication.

## Conclusion

In this study, ORF1a nsp5 P108S, S protein D614G, ORF3a Q57H and N protein P151L mutations have been predicted to alter their structures and/or functions. Since all the reported variants of concern contain multiple mutations present in multiple SARS-CoV-2 proteins, it is necessary to evaluate the impact of these mutations in combination on viral transmission and pathogenicity. The biological consequences of these nonsynonymous mutations of SARS-CoV-2 proteins should be further validated with *in vivo* and *in vitro* experimental studies in the future.

## Ethics and dissemination

No ethical approval is required for data analysis in this study (EA0802021).

## Author contribution

CHN contributes to the concept, design, supervision of the project. SBZ, WXB, YYY and SK contribute to the design, methodology, and data collection. SBZ, WXB, YYY and SK contributed to the analysis, and interpretation of data.

All authors were involved in drafting and revising the manuscript and approved the final version.

## Data and software availability

SARS-CoV-2 virus genome sequence data were downloaded from the GISAID Database. The additional multiple alignment data can be obtained from FigShare

Figshare: MSA (SARS-CoV-2). https://doi.org/10.6084/m9.figshare.16681900.v4 ^33^

This project contains the following underlying data.

• MSA_0 (31-12-2019 to 31-05-2020).fasta file contains multiple sequence alignment data of SARS-CoV-2 genome sequences ranging between 31-12-2019 and 31-05-2020.
• MSA_1 (01-06-2020 to 15-10-2020).fasta file contains multiple sequence alignment data of SARS-CoV-2 genome sequences ranging between 01-06-2020 and 15-10-2020.
• MSA_2 (16-10-2020 to 31-01-2021).fasta file contains multiple sequence alignment data of SARS-CoV-2 genome sequences ranging between 16-10-2020 and 31-01-2021.
• MSA_3 (01-02-2021 to 22-03-2021).fasta file contains multiple sequence alignment data of SARS-CoV-2 genome sequences ranging between 01-02-2021 to 22-03-2021.

Data are available under the terms of the Creative Commons Attribution 4.0 International license (CC-BY 4.0).

The python script used for the identification of SARS-CoV-2 genome mutations can be obtained through GitHub ( https://github.com/wxboon98/Mutations-Identification).

The same set of SARS-CoV-2 genomic data was also used to perform multiple sequence alignment analysis to identify the SARS-CoV-2 genomic mutations for another paper, titled “Prediction of the Effects of Synonymous Variants on SARS-CoV-2 Genome”. ^34^ MEGA-X software was used to determine if the mutations are synonymous or nonsynonymous mutations for the subsequent prediction and other analyses.
