# Supplementary material for: Prediction of the effects of the top 10 nonsynonymous variants from 30229 SARS-CoV-2 strains on their proteins
Source: F1000Res. 2022 May 18;11:9. Originally published 2022 Jan 6. [Version 2] doi: 10.12688/f1000research.72904.2 (PMC9184924; doi:10.12688/f1000research.72904.2)
Supplement: Peer review history

# Peer review: Reviewer response for version 2

**DOI:** 10.5256/f1000research.133846.r138228

**Published:** 2022-06-09

**Authors:** Ujwal Ranjit Bagal, Vishal Nayak

**Affiliations:** 1 Centers for Disease Control and Prevention, Atlanta, GA, USA; 2 Frederick National Laboratory for Cancer Research, Frederick, MD, USA

The authors have addressed all our concerns in the revised version of the manuscript. The current version looks almost ready for indexing. 

 Below are a few grammatical errors we want them to correct or rephrase the sentence.

1. Since N protein R203 and G204 are located at a disordered region which does not have a .....
  Rephrase the sentence.
2. A predicted model of N protein model (QHD43423, e.....
  Rephrase the sentence
3. Inappropriate sequences of base calling errors, “N” unresolved nucleotides, and undefinable gaps were omitted.
  with basecalling errors
4. Hence, it is not surprised that five nonsynonymous mutations, including ORF1b nsp12 P323L, S protein N501Y, S protein D614G, N protein R203K and N protein G204R identified in this study were also part of the defining mutations in the alpha variant
  change to surprising instead of surprised

Is the work clearly and accurately presented and does it cite the current literature?

Partly

If applicable, is the statistical analysis and its interpretation appropriate?

Not applicable

Are all the source data underlying the results available to ensure full reproducibility?

Yes

Is the study design appropriate and is the work technically sound?

Partly

Are the conclusions drawn adequately supported by the results?

Partly

Are sufficient details of methods and analysis provided to allow replication by others?

No

Reviewer Expertise:

Genomics & Evolutionary Biology

We confirm that we have read this submission and believe that we have an appropriate level of expertise to confirm that it is of an acceptable scientific standard.

# Peer review: Reviewer response for version 2

**DOI:** 10.5256/f1000research.133846.r138227

**Published:** 2022-05-27

**Authors:** In-Hee Lee

**Affiliations:** 1 Computational Health Informatics Program, Boston Children's Hospital, Boston, MA, USA

The revised article properly addresses the points raised by the reviewers.

Is the work clearly and accurately presented and does it cite the current literature?

Yes

If applicable, is the statistical analysis and its interpretation appropriate?

Not applicable

Are all the source data underlying the results available to ensure full reproducibility?

Yes

Is the study design appropriate and is the work technically sound?

Partly

Are the conclusions drawn adequately supported by the results?

Yes

Are sufficient details of methods and analysis provided to allow replication by others?

Partly

Reviewer Expertise:

Genomics

I confirm that I have read this submission and believe that I have an appropriate level of expertise to confirm that it is of an acceptable scientific standard.

# Peer review: Reviewer response for version 1

**DOI:** 10.5256/f1000research.76515.r125647

**Published:** 2022-03-10

**Authors:** In-Hee Lee

**Affiliations:** 1 Computational Health Informatics Program, Boston Children's Hospital, Boston, MA, USA

The authors examined 10 selected nonsynonymous mutations in SARS-CoV-2 genome for their predicted effect on protein stability as well as their co-mutations. Overall the paper is written well to understand the experiments and analysis results.

**Methods:**

 1. Authors mentioned that there were 231 nonsynonymous mutations from the 30,229 SARS-CoV-2 genome sequences used in the analysis. However, only 10 were intensively investigated throughout the paper. Can you explain the criteria why these mutations were selected?

 2. The number of nonsynonymous mutations in coding sequences (Figure 1) may need to be adjusted by the length of each coding sequence.

**Results:**

 3. Given the diverse nature of sequences collected by GISAID, it would be helpful to understand if authors could provide more details about 30,229 sequences used in the study: geographic information for the origin of collection, genetic nomenclature (Nextstrain clade, PANGO lineage, variants of concern or interest by WHO).

 4. Co-mutation analysis was particularly intriguing because the co-mutation frequencies were high for most mutations. Can you discuss more about this in the Discussion? Also, I wonder if it will persist when co-mutation analysis were done by genetic nomenclature.

 5. Mutations are as both nucleotide changes and amino acid changes in most figures and tables, but Figure 2 only shows nucleotide changes while Table 4 and 5 show only amino acid changes. Can you put amino acid changes on Figure 2 for easy cross-match with other figures and tables?

**Others:**

 6. Specifying 10 nonsynonymous variants in the title may help readers from misinterpreting that the paper conducted an intensive investigation of all possible nonsynonymous variants.

 7. The findings reported by the paper might have been limited to the sequences collected from a time-period almost a year ago (2020-01-01 ~ 2021-03-21). Adding discussion about the impact of the study with the advent of omicron variants would be interesting to readers of wide backgrounds.

Is the work clearly and accurately presented and does it cite the current literature?

Yes

If applicable, is the statistical analysis and its interpretation appropriate?

Not applicable

Are all the source data underlying the results available to ensure full reproducibility?

Yes

Is the study design appropriate and is the work technically sound?

Partly

Are the conclusions drawn adequately supported by the results?

Yes

Are sufficient details of methods and analysis provided to allow replication by others?

Partly

Reviewer Expertise:

Genomics

I confirm that I have read this submission and believe that I have an appropriate level of expertise to confirm that it is of an acceptable scientific standard, however I have significant reservations, as outlined above.

# Response

**Published:** 2022-05-09

**Authors:** Chong Han Ng

**Affiliations:** Multimedia University, Malaysia

The authors examined 10 selected nonsynonymous mutations in SARS-CoV-2 genome for their predicted effect on protein stability as well as their co-mutations. Overall the paper is written well to understand the experiments and analysis results.

 Methods:

 1. Authors mentioned that there were 231 nonsynonymous mutations from the 30,229 SARS-CoV-2 genome sequences used in the analysis. However, only 10 were intensively investigated throughout the paper. Can you explain the criteria why these mutations were selected?

**RE:** The 10 nonsynonymous mutations are identified based on the mutations with the top 10 highest frequency identified from this study.

 2. The number of nonsynonymous mutations in coding sequences (Figure 1) may need to be adjusted by the length of each coding sequence.

**RE:** Figure 1 shows the total number of the mutations in each gene, eg D614G, N501Y in S protein. It is not relevant to include extra information.

 Results:

 3. Given the diverse nature of sequences collected by GISAID, it would be helpful to understand if authors could provide more details about 30,229 sequences used in the study: geographic information for the origin of collection, genetic nomenclature (Nextstrain clade, PANGO lineage, variants of concern or interest by WHO).

**RE:** We included the information on primary lineages for the past and present VOCs associated with the top 10 nonsynonymous mutations and their mutation frequency in Table 3. We also included the information on the geographical distribution of the SARS-CoV-2 dataset with the date range summarized in a table as extended data. While we can see diverse genome dataset coming from different regions, we don’t know if there is a good correlation between the reported COVID case number and the number of SARS-CoV-2 genome data deposited to GISAID database. There can be some disparity in genomic surveillance in different countries due to these possible reasons, such as the quality of the sequencing data, the accessibility to research funding resource, the socioeconomic status, the government policy. Therefore, it is less relevant for our study since we are not aimed to monitor the SARS-CoV-2 mutation profile in different regions.

 4. Co-mutation analysis was particularly intriguing because the co-mutation frequencies were high for most mutations. Can you discuss more about this in the Discussion? Also, I wonder if it will persist when co-mutation analysis were done by genetic nomenclature.

**RE:** The GESS database we used does not support co-mutation analysis by the clades or lineages. If we use other tools, we may get different results. Therefore, we didn’t do co-mutation analysis by the clades or lineages. However, we expand the discussion part on co-mutation analysis based on the information of mutation frequency percentage by the lineages derived from COVID CG database.

 5. Mutations are as both nucleotide changes and amino acid changes in most figures and tables, but Figure 2 only shows nucleotide changes while Table 4 and 5 show only amino acid changes. Can you put amino acid changes on Figure 2 for easy cross-match with other figures and tables?

**RE:** Figure 2 has been revised with the additional information on amino acid changes.

 Others:

 6. Specifying 10 nonsynonymous variants in the title may help readers from misinterpreting that the paper conducted an intensive investigation of all possible nonsynonymous variants.

**RE:** To reflect the scope of the study better, the title of paper has been revised to “Prediction of the effects of the top 10 nonsynonymous variants from 30229 SARS-CoV-2 strains on their proteins.”

 7. The findings reported by the paper might have been limited to the sequences collected from a time-period almost a year ago (2020-01-01 ~ 2021-03-21). Adding discussion about the impact of the study with the advent of omicron variants would be interesting to readers of wide backgrounds.

**RE:** Different SARS-CoV-2 variants of concern have specific sets of defining mutations; some are common among these VOCs while some are unique. Additional paragraph in the discussion section has been added to discuss the impact of our study and to explain why the study of some of the identified mutations remain relevant for the newer variants.

# Peer review: Reviewer response for version 1

**DOI:** 10.5256/f1000research.76515.r123168

**Published:** 2022-02-28

**Authors:** Ujwal Ranjit Bagal, Vishal Nayak

**Affiliations:** 1 Centers for Disease Control and Prevention, Atlanta, GA, USA; 2 Frederick National Laboratory for Cancer Research, Frederick, MD, USA

The authors in this paper have tried to use an extensive set of SARS-COV-2 genomes to identify and select the top 10 non-synonymous mutations for analyzing the co-mutation effect, as well as its effect on the stability and flexibility of the protein structure. Overall, the paper is well written in terms of language and grammar, experiments were well conducted. The title suggests all the variants were analyzed. Hence, it can be modified to show that only the top 10 non-synonymous mutations were studied. Abstract is well written giving all details of the papers finding.

 SARS-COV-2 is an excellent example where there are public repositories with highly curated whole genome datasets and associated metadata are available for analysis. The authors need to use more data from 2020 to 2021 as the number of curated genomes available in GISAID is large (200,000+). This will make the analysis less obsolete. 

 The authors have applied meta-prediction methods for variant analysis. If relevant data in terms of genetic clade, and region where the sample was collected from, can be added to this analysis the results will be more relevant and can be verified using laboratory techniques. Hence, we suggest the authors try to incorporate these points and resolve a few issues mentioned below and resubmit the paper with additional tables and content.

 Below are a few comments for the authors we as reviewers suggest:

1. The introduction seems too small. More details about the virus and work showing the effect of non-synonymous mutations affecting the viral efficacy need to be mentioned.
2. In the Methods section
  1. For the downloaded datasets, what was the threshold used for coverage? A table showing the number of genomes with date (range should do), coverage above threshold, genetic nomenclature (clade name), and geographical information will be helpful to understand the diversity within the dataset.
  2. You have performed a Co-mutation analysis using the GESS database. Does it provide information about the mutation frequency, which genetic nomenclature it was observed? If you can provide that information, it will be useful. The concurrence table is good, but with knowledge of the above information it will become more relevant.
  3. What was the criteria used for “top 10 nonsynonymous mutations”?
  4. For prediction of mutation effect on protein function, where was the GTF file, as well as the VCF files, obtained from? There is no mention of whole genome SNP analysis. This part is a bit confusing. Clarification is required.
  5. “Mutations with a value less than-2.5 were considered as deleterious”. Can you provide a reference showing why -2.5 is used as a threshold? Same with the SIFT 4G score threshold of 0.05.
3. In the Results section:
  1. Figure 2: Is it possible to add the amino acid changes (e.g., D614G) instead of just nucleotide mutations for better understanding?
  2. Is it possible to show the genetic nomenclature associated with the top 10 nonsynonymous mutations?
  3. Also, if possible, can you add figures showing the domain or the position on a 3D protein structure? This is optional as you have discussed the domain for few proteins in the discussion section.
4. In the Discussion section:
  1. You write “showed very high concurrence ratio with other mutations since they emerged in the early phase of the pandemic”. How did you come to this conclusion? With reference to our comments in the results and methods section, if you can add this information in a tabular format it will be more informative.
  2. “The combination of both mutations may enhance viral fitness based on epidemiological data”. There is no mention of the epidemiological data in the results section. If it's in the supplementary files, mention it.
  3. “P108S mutation diminished its activity, possibly leading to a reduction in disease severity.” Can you mention in which genetic clade it was observed?

Is the work clearly and accurately presented and does it cite the current literature?

Partly

If applicable, is the statistical analysis and its interpretation appropriate?

Not applicable

Are all the source data underlying the results available to ensure full reproducibility?

Yes

Is the study design appropriate and is the work technically sound?

Partly

Are the conclusions drawn adequately supported by the results?

Partly

Are sufficient details of methods and analysis provided to allow replication by others?

No

Reviewer Expertise:

Genomics & Evolutionary Biology

We confirm that we have read this submission and believe that we have an appropriate level of expertise to confirm that it is of an acceptable scientific standard, however we have significant reservations, as outlined above.

# Response

**Published:** 2022-05-09

**Authors:** Chong Han Ng

**Affiliations:** Multimedia University, Malaysia

*"The authors in this paper have tried to use an extensive set of SARS-COV-2 genomes to identify and select the top 10 non-synonymous mutations for analyzing the co-mutation effect, as well as its effect on the stability and flexibility of the protein structure. Overall, the paper is well written in terms of language and grammar, experiments were well conducted. The title suggests all the variants were analyzed. Hence, it can be modified to show that only the top 10 non-synonymous mutations were studied. Abstract is well written giving all details of the papers finding."*

**RE: **To reflect the scope of the study better, the title of paper has been revised to “Prediction of the effects of the top 10 nonsynonymous variants from 30229 SARS-CoV-2 strains on their proteins.”

*"SARS-COV-2 is an excellent example where there are public repositories with highly curated whole genome datasets and associated metadata are available for analysis. The authors need to use more data from 2020 to 2021 as the number of curated genomes available in GISAID is large (200,000+). This will make the analysis less obsolete."*

**RE:** We used SARS-CoV-2 virus genome data ranging from 1st January 20 to 22 March 21. The first draft of the manuscript was prepared in late June 21 and submitted in late August 21. Due to some delay in the editorial review, the paper was only published on 6th January 2022. Five nonsynonymous mutations, including ORF1b nsp12 P323L, S protein N501Y, S protein D614G, N protein R203K and N protein G204R identified in this study were also part of the defining mutations in the alpha variant. The mutational profile of SARS-CoV-2 genome is changing very rapidly. However, we are not aiming to monitor the mutational changes of SARS-CoV-2 genome since it is impossible to keep up with the exponential growth of these data. As of 26 April 2022, there are more than 10 million SARS-CoV-2 virus genomes sequences deposited in GISAID database. Although it is useful to get more recent dataset, it is out of the scope of our study to update the data. Both identification of the SARS-CoV-2 mutations and the prediction analysis of the biological consequences of these mutations are very time-consuming processes. Additional paragraph in the discussion section has been added to discuss the impact of our study and to explain why the study of some of the identified mutations remain relevant for the newer variants.

*"The authors have applied meta-prediction methods for variant analysis. If relevant data in terms of genetic clade, and region where the sample was collected from, can be added to this analysis the results will be more relevant and can be verified using laboratory techniques. Hence, we suggest the authors try to incorporate these points and resolve a few issues mentioned below and resubmit the paper with additional tables and content."*

**RE: ** We included the information on primary lineages for the past and present VOCs associated with the top 10 nonsynonymous mutations and their mutation frequency in Table 3. We also included the information about the geographical distribution of the SARS-CoV-2 dataset with the date range summarized in a table as extended data. We are not performing extra experiments to verify the prediction data since our work is primarily focused on prediction analysis of the mutations. The lab work is out of the scope of this paper, and it is too time-consuming and labour-intensive to perform experimental works.

*"Below are a few comments for the authors we as reviewers suggest:*

*The introduction seems too small. More details about the virus and work showing the effect of non-synonymous mutations affecting the viral efficacy need to be mentioned."*

**RE:** The introduction section with the examples of the effect of non-synonymous mutations affecting the viral efficacy has been included in the last paragraph.

*"In the Methods section*

*For the downloaded datasets, what was the threshold used for coverage? A table showing the number of genomes with date (range should do), coverage above threshold, genetic nomenclature (clade name), and geographical information will be helpful to understand the diversity within the dataset."*

**RE:** For the downloaded dataset, high coverage filter has been applied. The high coverage is defined as only entries with <1% Ns and <0.05% unique amino acid mutations (not seen in other sequences in database) and no insertion/deletion unless verified by submitter, according to GISAID. We included the information on primary lineages for the past and present VOCs associated with the top 10 nonsynonymous mutations and their mutation frequency in Table 3.  We have the information about the geographical distribution of the SARS-CoV-2 dataset with the date range summarized in a table as extended data. While we observe a diverse genome dataset coming from different regions, we don’t know if there is a good correlation between the reported COVID case number and the number of SARS-CoV-2 genome data deposited to GISAID database. There may be some disparity in genomic surveillance in different countries due to these possible reasons, such as the quality of the sequencing data, the accessibility to the research funding resource, the socioeconomic status, the government policy. Therefore, it is less relevant for our study since we are not aimed to monitor the SARS-CoV-2 mutation profile in different regions.

*"You have performed a Co-mutation analysis using the GESS database. Does it provide information about the mutation frequency, which genetic nomenclature it was observed? If you can provide that information, it will be useful. The concurrence table is good, but with knowledge of the above information it will become more relevant."*

**RE:** The GESS database doesn’t have the information about the mutation frequency of the mutations associated with the lineages or clades. However, we included the information obtain from COVID CG database on primary lineages for the past and present VOCs associated with the top 10 nonsynonymous mutations and their mutation frequency in Table 3. In addition, we expand the discussion part on co-mutation analysis based on the information of mutation frequency percentage by the lineages.

*"What was the criteria used for “top 10 nonsynonymous mutations”?"*

**RE:** The top 10 nonsynonymous mutations are identified based on the mutations with the highest frequency identified from this study.

*"For prediction of mutation effect on protein function, where was the GTF file, as well as the VCF files, obtained from? There is no mention of whole genome SNP analysis. This part is a bit confusing. Clarification is required."*

**RE:**  Additional information on GTF and VCF files are added in the methods. The GTF and VCF files are deposited in Figshare and the related information are included in Data and software availability section. It is a whole genome SNP analysis, but the SIFT 4G results of ORF1b nsp12 P323L and A423V, S protein N501Y and N protein P151L mutations cannot be obtained due to missing data in the Ensembl database. 

*“Mutations with a value less than-2.5 were considered as deleterious”. Can you provide a reference showing why -2.5 is used as a threshold? Same with the SIFT 4G score threshold of 0.05.*

**RE:**  Both references for the scoring method of SIFT4G and PROVEAN have been included.

 In the Results section:

*"Figure 2: Is it possible to add the amino acid changes (e.g., D614G) instead of just nucleotide mutations for better understanding?"*

**RE:** Figure 2 has been revised with the additional information on amino acid changes.

*"Is it possible to show the genetic nomenclature associated with the top 10 nonsynonymous mutations?"*

**RE:** The information on the mutation frequency percentage in the primary lineages associated with the past and present variant of concern (VOC) have been updated in Table 3.

*"Also, if possible, can you add figures showing the domain or the position on a 3D protein structure? This is optional as you have discussed the domain for few proteins in the discussion section."*

**RE:** There are multiple SARS-CoV-2 proteins mentioned in the paper. We are not doing any work on protein structure modelling. The readers should refer to Protein Data Bank if they want to know more specific information on the protein domains.

*"In the Discussion section:*

*You write “showed very high concurrence ratio with other mutations since they emerged in the early phase of the pandemic”. How did you come to this conclusion? With reference to our comments in the results and methods section, if you can add this information in a tabular format it will be more informative."*

**RE:** Table 3 shows that S protein D614G and ORF1b nsp12 P323L mutations have the top 2 highest frequency. They are found in more than 90% of 30229 SARS-CoV-2 genome sequences, which are from the early batch of SARS-CoV-2 genome data. A similar finding has been reported by S. Ilmjärv et al., “Concurrent mutations in RNA-dependent RNA polymerase and spike protein emerged as the epidemiologically most successful SARS-CoV-2 variant,” Sci. Rep., vol. 11, no. 1, pp. 1–13, Jul. 2021.

*“The combination of both mutations may enhance viral fitness based on epidemiological data”. There is no mention of the epidemiological data in the results section. If it's in the supplementary files, mention it.*

**RE**: We are referring to the study published by S. Ilmjärv et al., “Concurrent mutations in RNA-dependent RNA polymerase and spike protein emerged as the epidemiologically most successful SARS-CoV-2 variant,” *Sci. Rep*., vol. 11, no. 1, pp. 1–13, Jul. 2021.

*“P108S mutation diminished its activity, possibly leading to a reduction in disease severity.” Can you mention in which genetic clade it was observed?*

**RE:** The genetic clade associated with ORF1a nsp5 P108S is 20B-T (lineage B.1.1.284). However, it is unknown if this mutation is associated with the past and current of variants of concern since the data is unavailable from COVID CG database.
